# Laboratory indicators combined with liver function scores for predicting suboptimal hepatobiliary phase imaging during gadoxetic acid-enhanced MRI

**DOI:** 10.3389/fmed.2026.1945459

**Published:** 2026-10-13

**Authors:** Fuhe Qiu, Fuling Huang, Haihui Pan, Jiancong Li, Feichen Lu

**Affiliations:** Department of Medical Imaging, Guangxi Medical University Cancer Hospital, Nanning, China

**Keywords:** albumin-bilirubin score, contrast-enhanced magnetic resonance imaging, gadoxetic acid, hepatobiliary phase, image quality, laboratory indicators, liver function scores

## Abstract

**Background:**

Suboptimal hepatobiliary-phase (HBP) enhancement may limit the diagnostic quality of gadoxetic acid-enhanced MRI. We investigated whether routinely available laboratory indicators and liver function scores could identify patients at increased risk of suboptimal HBP imaging.

**Methods:**

This retrospective study included 555 patients with focal liver lesions who underwent gadoxetic acid-enhanced MRI, including 460 with good and 95 with suboptimal HBP image quality. Associations between laboratory indicators, liver function scores, and HBP image quality were evaluated using correlation and logistic regression analyses. Model discrimination, calibration, internal validation, and potential clinical utility were assessed using ROC analysis, bootstrap resampling, calibration analysis, and decision-curve analysis.

**Results:**

The ALBI score (OR=2.391, 95% CI: 1.364–4.190), GGT (OR=1.003, 95% CI: 1.001–1.004), TBA (OR=1.010, 95% CI: 1.003–1.017), and Hb (OR=0.982, 95% CI: 0.971–0.993) were independently associated with suboptimal HBP imaging. The combined model yielded an AUC of 0.758 (95% CI: 0.701–0.816), with 63.16% sensitivity and 78.70% specificity. Bootstrap internal validation that repeated the entire model-development procedure yielded a mean AUC optimism of 0.044 and an optimism-corrected AUC of 0.714, indicating that moderate discrimination was retained after correction for model-development optimism. The apparent and optimism-corrected Brier scores were 0.116 and 0.128, respectively.

**Conclusions:**

A model combining the ALBI score, GGT, TBA, and Hb showed moderate discrimination for suboptimal HBP imaging. These routinely available indicators may assist pre-MRI risk stratification, but external validation is required before clinical implementation.

## Highlights

- ALBI score, GGT, TBA, and Hb were independently associated with suboptimal hepatobiliary phase imaging.
- The combined model retained moderate discrimination after bootstrap correction of the complete model-development procedure.
- LPC showed predominantly weak correlations with liver function scores and laboratory indicators.
- Routine laboratory indicators may help identify patients at risk for suboptimal gadoxetic acid-enhanced MRI.

## 1 Background

Focal liver lesions are common hepatic diseases in clinical practice and their detection, differentiation between benign and malignant lesions, treatment planning, and therapeutic response evaluation rely on accurate imaging assessment (1). Magnetic resonance imaging (MRI) offers several advantages in the diagnosis of liver diseases, including high soft-tissue resolution, multiple sequences, multiparametric imaging, and multiplanar acquisition (2). Gadoxetic acid disodium (Gd-EOB-DTPA) is a hepatobiliary-specific MRI contrast agent that provides conventional dynamic contrast-enhanced imaging information and reflects hepatocyte uptake and biliary excretion through hepatobiliary phase imaging (3). Under normal conditions, hepatocytes take up Gd-EOB-DTPA through organic anion-transporting polypeptides (OATPs), resulting in a marked enhancement of the liver parenchyma during the hepatobiliary phase, thereby improving the detection rate and diagnostic accuracy of liver lesions (4, 5). Therefore, Gd-EOB-DTPA-enhanced MRI has been widely used for the imaging evaluation of focal liver lesions.

However, in some patients with impaired liver function or biliary excretion disorders, the decreased hepatocyte uptake and transport capacity may lead to insufficient liver parenchymal enhancement and poor bile duct visualization during the hepatobiliary phase, resulting in reduced image quality and affecting lesion detection and diagnostic decision-making (6, 7). Failure to identify the risk of suboptimal imaging before the examination in such patients may result in ineffective or inefficient hepatobiliary phase scanning, increase the examination time and medical costs, and impose an unnecessary burden on patients (8). Therefore, predicting the image quality of the hepatobiliary phase in Gd-EOB-DTPA-enhanced MRI before examination is of great significance for optimizing imaging protocols, improving examination efficiency, and enhancing the clinical workflow (9).

At present, the indocyanine green clearance test is considered an important method for evaluating hepatic functional reserve. The indocyanine green retention rate at 15 min (ICG-R15) and indocyanine green clearance rate (ICG-K) reflect hepatocyte uptake, transport, and biliary excretion function and are correlated with the degree of liver enhancement and biliary excretion (10). However, this test is relatively complex to perform and is limited by equipment and workflow requirements. Therefore, it is not routinely performed before MRI in all patients, which limits its clinical application (11). In contrast, laboratory indicators and liver function scores are readily available, inexpensive, and widely used in clinical practice, making them common tools for assessing liver function status and cholestasis (12). The albumin-bilirubin (ALBI) and the Model for End-Stage Liver Disease (MELD) scores reflect hepatic functional reserve and liver disease severity from different perspectives (13) In addition, total bilirubin (TBIL), direct bilirubin (DBIL), total bile acid (TBA), gamma-glutamyl transferase (GGT), alkaline phosphatase (ALP), albumin (ALB), and hemoglobin (Hb) may also be associated with the hepatobiliary uptake and excretion of Gd-EOB-DTPA (14).

Therefore, the present study retrospectively analyzed data from patients with focal liver lesions who underwent Gd-EOB-DTPA-enhanced MRI. The baseline characteristics, laboratory indicators, and liver function scores were compared between the good and suboptimal image quality groups. The relationships between these indicators and the hepatobiliary phase image quality were further analyzed. Their predictive value for suboptimal hepatobiliary-phase imaging was evaluated to explore whether routinely available laboratory indicators and liver function scores could support pre-MRI risk stratification.

## 2 Methods

### 2.1 Study design and patients

This retrospective study included 555 patients with focal liver lesions who underwent gadoxetic acid disodium-enhanced MRI at Guangxi Medical University Cancer Hospital between March 2024 and December 2025.

The inclusion criteria were completion of hepatobiliary phase imaging with Gd-EOB-DTPA-enhanced MRI, complete clinical and laboratory data, and no history of hepatic or splenic surgery. The exclusion criteria were severe respiratory motion artifacts affecting the accuracy of image measurements; inaccurate hepatobiliary phase delay time, defined as a deviation of more than 5 min from the standard 20-min hepatobiliary phase delay; incomplete laboratory test results; diffuse liver lesions, defined as more than five hepatic lesions; a large focal liver lesion with a single lesion diameter >3 cm that could affect image measurements; extensive abnormal enhancement of the intrahepatic parenchyma during the arterial or portal venous phase; and focal or diffuse fatty liver disease or intrahepatic iron deposition. Patients with missing clinical or laboratory variables required for the primary analyses were excluded before model development, and no statistical imputation was performed. Accordingly, the final analytic cohort represented a complete-case dataset. Severe motion artifacts, fatty liver disease, and intrahepatic iron deposition were excluded because these conditions can directly affect image quality or hepatic signal-intensity measurements independently of hepatobiliary contrast uptake and excretion. These exclusions were therefore intended to reduce technical and tissue-related interference with the assessment of hepatobiliary-phase enhancement.

This study was approved by the Ethics Committee of Guangxi Medical University Cancer Hospital (approval no. KY2024536). The requirement for written informed consent was waived by the Ethics Committee because of the retrospective nature of the study.

### 2.2 MRI examination protocol

All patients underwent MRI using a uMR 790 3.0-T MRI system (United Imaging Healthcare, Shanghai, China) with a 16-channel phased-array body coil. Patients were examined in the supine position, with the scanning range extending from the diaphragmatic dome to the inferior margin of the liver. The imaging protocol included axial T1-weighted imaging (T1WI), T2-weighted imaging (T2WI), fat-suppressed T2WI using spectral attenuated inversion recovery (T2WI-SPAIR), diffusion-weighted imaging (DWI), and dynamic contrast-enhanced T1WI. T2WI was acquired using a fast spin-echo sequence, T2WI-SPAIR using a fat-suppressed fast spin-echo sequence, and axial T1WI using a fat-suppressed gradient-echo sequence based on water–fat separation.

Before image acquisition, patients underwent respiratory training and were instructed to inhale deeply, exhale slowly and steadily to end expiration, and then hold their breath at end expiration during image acquisition. The acquisition time for each breath-hold acquisition was 15 s. The T1WI parameters were as follows: repetition time (TR), 3.88 ms; echo time (TE), 1.38 ms; flip angle, 12°; slice thickness, 5 mm; slice interpolation factor, 1.67; matrix, 304  ×  182; and field of view (FOV), 400  ×  300 mm. The T2WI parameters were: TR, 4,000 ms; TE, 89.4 ms; slice thickness, 5 mm; interslice gap, 1 mm; matrix, 320  ×  320; and FOV, 380  ×  380 mm. The T2WI-SPAIR parameters were: TR, 3,676 ms; TE, 82.2 ms; slice thickness, 5 mm; interslice gap, 1 mm; matrix, 320  ×  320; and FOV, 380  ×  380 mm. The DWI parameters were: TR, 4,000 ms; TE, 65 ms; slice thickness, 5 mm; interslice gap, 1 mm; matrix, 144 × 130; FOV, 380 × 380 mm; and b values of 50 and 800 s/mm^2^.

Contrast-enhanced imaging, including hepatobiliary-phase acquisition, was performed using a three-dimensional gradient-echo sequence with the following parameters: TR, 3.88 ms; TE, 1.38 ms; flip angle, 10°; slice thickness, 5 mm; slice interpolation factor, 1.67; matrix, 304 × 182; and FOV, 400 × 300 mm. Gd-EOB-DTPA was administered through an antecubital vein at a dose of 25 μmol/kg (equivalent to 0.1 mL/kg) with an injection rate of 1 mL/s, followed by a 10-mL saline flush. Arterial, portal venous, transitional, and hepatobiliary-phase images were acquired at 16–32 s, 90 s, 180 s, and 20 min after contrast-agent administration, respectively. All MRI examinations and image acquisitions were performed by supervising technologists who had undergone standardized departmental training.

### 2.3 Laboratory parameters and liver function score calculation

Laboratory measurements obtained within 14 days before the MRI examination were collected from the electronic medical records, including TBIL, GGT, international normalized ratio (INR), platelet count (PLT), TBA, DBIL, IBIL, alkaline phosphatase (ALP), creatinine (Cr), aspartate aminotransferase (AST), alanine aminotransferase (ALT), Hb, ALB, and serum ferritin (SF). The precise time interval between laboratory testing and the MRI examination was recorded for each patient; the median interval was 5 days. When multiple laboratory measurements were available within this interval, the measurement closest to the MRI examination was used. The ALBI and MELD scores were also calculated (12, 13).

The ALBI score was calculated using TBIL and ALB according to the following formula: ALBI score = 0.66×log10 [TBIL (*μ*mol/L)]−0.085 × [ALB (g/L)]. A higher ALBI score indicates a poorer hepatic functional reserve. The MELD score used in this study was the traditional (original) MELD score, which was calculated from TBIL, INR, and Cr according to the following formula: MELD score = 3.8×ln [TBIL (mg/dL)] + 11.2×ln (INR) + 9.6×ln [Cr (mg/dL)] + 6.4×etiology, where TBIL and Cr are entered in mg/dL, INR is a dimensionless ratio, and the etiology term was assigned a value of 0 for cholestatic or alcoholic liver disease and 1 for all other etiologies (15). A higher MELD score indicates more severe liver dysfunction and a higher risk of liver disease.

### 2.4 Image analysis and image quality assessment

For objective image quality assessment, two physicians placed regions of interest (ROIs) in the right, left, and caudate lobes at the hepatic hilum level on the hepatobiliary phase images. ROIs were placed on a picture archiving and communication system (PACS) workstation and were manually adjusted to avoid visible intrahepatic vessels, bile ducts, focal liver lesions, imaging artifacts, and regions of partial-volume averaging. The hepatic parenchymal ROIs ranged in size from 1.0 to 2.0 cm2 (100–200 mm2), and the portal vein ROIs ranged from 8 to 40 mm2 depending on the diameter of the vessel. The signal intensity of the liver parenchyma in each region was measured and the mean value was recorded as the mean liver parenchymal signal intensity (SI_liver). The signal intensity of the intrahepatic portal vein was measured at the bifurcation level of the left and right portal veins and the mean value was recorded as the mean portal vein signal intensity (SI_portal vein). Measurements were performed by one physician and were independently reviewed by a second physician; when the two physicians disagreed regarding ROI placement or the measured signal intensities, the final value was determined by consensus together with a third senior radiologist. Both physicians were blinded to the patients' laboratory results, liver function scores, and clinical data during ROI placement and measurement. The liver parenchyma-to-portal vein contrast ratio (LPC) was calculated using the following formula: LPC = SI_liver/SI_portal vein. LPC was used to quantitatively evaluate liver parenchymal enhancement during the hepatobiliary phase of gadoxetic acid disodium-enhanced MRI (16). A representative example of ROI placement and signal intensity measurement on hepatobiliary phase images is shown in Figure 1.

**Figure 1:**
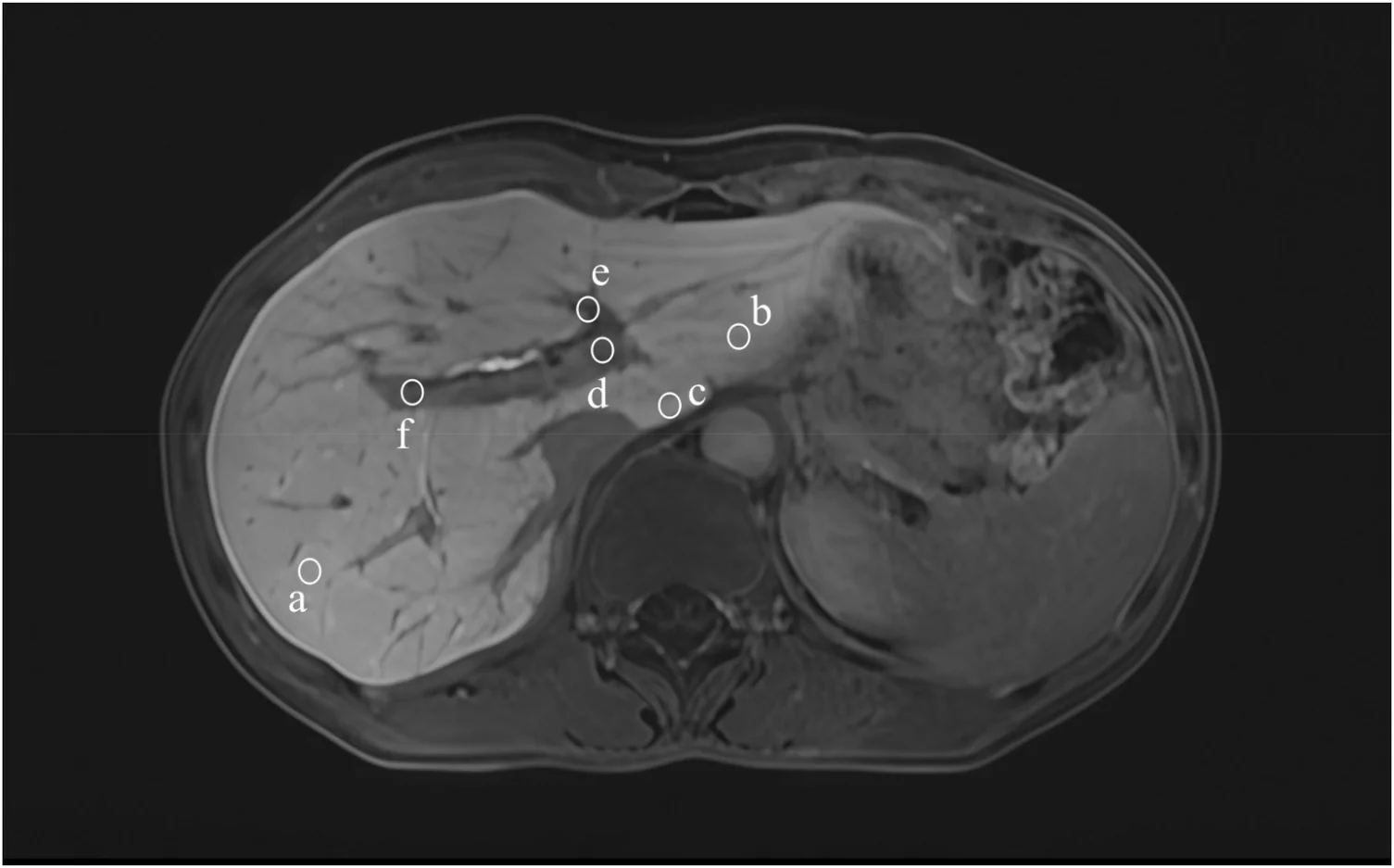
ROI placement and signal intensity measurement on hepatobiliary phase images. **(a)** ROI in the right liver lobe; **(b)** ROI in the left liver lobe; **(c)** ROI in the caudate lobe; **(d)** ROI in the main portal vein trunk; **(e)** ROI in the left portal vein branch; **(f)** ROI in the right portal vein branch. SI_liver was calculated as the mean signal intensity of hepatic ROIs, SI_portal vein was calculated as the mean signal intensity of portal venous ROIs, and LPC was defined as SI_liver/SI_portal vein.

For subjective image quality assessment, hepatobiliary phase images were independently evaluated in a blinded manner by two radiologists with 8 and 12 years of experience in abdominal MRI, respectively. Both radiologists were blinded to the patients' laboratory results, liver function scores, clinical data, and the results of the objective signal-intensity measurements. In cases of disagreement, the final assessment was determined by a third senior radiologist. Liver parenchymal enhancement and biliary contrast excretion are established components of hepatobiliary-phase image-quality assessment, including the functional liver imaging score framework (6, 17). Accordingly, two domains were evaluated in the present study: liver parenchymal signal intensity and common bile duct enhancement. Liver parenchymal signal intensity was scored as follows: 1 = the liver parenchymal signal was markedly higher than the intrahepatic vascular signal; 2 = the liver parenchymal signal was approximately equal to the intrahepatic vascular signal; and 3 = the liver parenchymal signal was markedly lower than the intrahepatic vascular signal. Common bile duct enhancement was scored as follows: 1 = marked enhancement compared with pre-contrast images with clearly visible margins; 2 = partial enhancement with blurred margins; and 3 = no visible enhancement. For the present analysis, image quality was operationally classified as good when both components received a score of 1, representing concurrent adequate liver parenchymal enhancement and clear biliary excretion. Images not meeting both criteria were classified as suboptimal. This composite classification was used as an operational definition of overall hepatobiliary-phase image quality in this study rather than as a previously validated lesion-specific diagnostic adequacy threshold. Based on this definition, 460 patients were classified as having good image quality and 95 as having suboptimal image quality.

### 2.5 Statistical analysis

Statistical analyses were performed using SPSS version 26.0 and MedCalc version 16.2.0. Additional statistical analyses required for model validation and performance assessment were performed using R software version 4.5.2 (R Foundation for Statistical Computing, Vienna, Austria). Continuous variables were first tested for normality using the Kolmogorov–Smirnov test. Normally distributed continuous variables are expressed as the mean ± standard deviation, and between-group comparisons were performed using the independent-samples t test. Non-normally distributed continuous variables are expressed as medians and interquartile ranges, and between-group comparisons were performed using the Mann–Whitney U test. Categorical variables are presented as numbers and percentages, and between-group comparisons were performed using the chi-square test or Fisher's exact test.

Spearman's correlation analysis was used to evaluate the correlations between clinical laboratory indicators, liver function scores, and hepatobiliary phase imaging parameters, including LPC, liver parenchymal signal score, and common bile duct enhancement score. The correlation coefficient r ranged from −1 to 1; |r|≥0.7 was considered a strong correlation, 0.3≤|r|<0.7 was considered a moderate correlation, and |r| < 0.3 was considered a weak correlation.

Interobserver agreement for the ordinal liver parenchymal signal and common bile duct enhancement scores was assessed using quadratic weighted Cohen's kappa, with 95% confidence intervals estimated by bootstrap resampling. Percent agreement between the two radiologists was also calculated.

Candidate predictors for the primary multivariable model were variables showing statistically significant between-group differences in the univariable analyses (*P* < 0.05), including age, BMI category, tumor history, ALBI score, MELD score, TBIL, GGT, INR, TBA, DBIL, IBIL, ALP, AST, Hb, ALB, and SF. Clinically relevant covariates not meeting the univariable screening criterion were evaluated separately in sensitivity analyses where appropriate. Because the ALBI and MELD scores incorporate laboratory variables that were also evaluated individually, multicollinearity was assessed before interpretation of the multivariable model using variance inflation factors (VIFs); generalized VIFs were used for categorical variables with more than two levels. Particular attention was paid to structural collinearity between composite liver function scores and their constituent variables. A forward stepwise logistic regression procedure was then used to derive a parsimonious prediction model, with entry and removal criteria of *P* = 0.05 and *P* = 0.10, respectively. Multicollinearity was also reassessed in the final model. To assess the robustness of the associations of the four predictors retained in the final model, a sensitivity analysis was performed by additionally adjusting for clinically relevant demographic and disease-related covariates available in the dataset, including age, BMI category, tumor history, liver cirrhosis, and hepatitis B virus infection. Detailed information on previous treatments and other underlying hepatic disease characteristics was not consistently available as structured variables in the retrospective records and therefore could not be included in the adjusted analysis. The adjusted odds ratios (ORs), 95% confidence intervals (CIs), and *P* values of the four primary predictors were estimated, and the discriminative performance of the adjusted model was evaluated using the AUC. A VIF >10 was considered indicative of substantial multicollinearity, whereas VIF values close to 1 indicated little evidence of collinearity. Receiver operating characteristic (ROC) curve analysis was used to evaluate the discrimination of individual predictors and the combined model for suboptimal hepatobiliary-phase imaging. The area under the curve (AUC), 95% confidence interval (CI), sensitivity, specificity, and Youden index were calculated. Optimal cutoff values were determined by maximizing the Youden index. Pairwise comparisons between the AUC of the combined model and those of the individual predictors were performed using the DeLong test for correlated ROC curves. Decision-curve analysis (DCA) was performed to evaluate the potential clinical utility of the combined model across a range of threshold probabilities. Net benefit was compared with the default strategies of treating all patients as being at risk of suboptimal imaging and treating no patients as being at risk. Internal validation of the entire model-development procedure was performed using 1,000 bootstrap resamples. Within each bootstrap sample, the same univariable screening procedures used in the primary analysis were repeated, and variables meeting the *P* < 0.05 screening criterion were entered into forward stepwise logistic regression using the same entry and removal criteria as in the primary analysis. The model derived within each bootstrap sample was evaluated both in that bootstrap sample and in the original cohort. Optimism in discrimination was defined as the difference between the bootstrap-sample AUC and the corresponding AUC obtained when the bootstrap-derived model was applied to the original cohort. Mean optimism across 1,000 resamples was subtracted from the apparent AUC of the original model to obtain the optimism-corrected AUC. Calibration was evaluated using the Brier score and a bootstrap optimism-corrected calibration curve. For the Brier score, the mean increase in prediction error from the bootstrap sample to the original cohort was added to the apparent Brier score to obtain an optimism-corrected estimate. All statistical tests were two-sided, and *P* < 0.05 was considered statistically significant.

## 3 Results

### 3.1 Comparison of baseline characteristics, liver function scores, and laboratory indicators between the two groups

A total of 638 patients with focal liver lesions who underwent gadoxetic acid-enhanced MRI between March 2024 and December 2025 were initially screened. Of these, 83 patients were excluded: 11 because of severe respiratory motion artifacts affecting image measurement, 42 because of incomplete laboratory data, 19 because of diffuse liver lesions (>5 lesions) or a large focal liver lesion (>3 cm) affecting image measurement, 4 because of extensive abnormal hepatic parenchymal enhancement during the arterial or portal venous phase, and 7 because of focal or diffuse fatty liver disease or intrahepatic iron deposition. No patient was excluded because of inappropriate hepatobiliary-phase timing. Finally, 555 patients were included in the analysis, including 460 with good hepatobiliary-phase image quality and 95 with suboptimal image quality (Figure 2). Significant differences were observed between the two groups in terms of age, BMI category, tumor history, ALBI score, MELD score, TBIL, GGT, INR, TBA, DBIL, IBIL, ALP, AST, Hb, ALB, and SF levels (all *P* < 0.05). However, there were no significant differences between the two groups in terms of sex, smoking history, long-term heavy alcohol consumption, history of previous gadoxetic acid disodium-enhanced MRI examination, liver cirrhosis, hepatitis B virus infection, COPD, diabetes mellitus, hypertension, and PLT, Cr, or ALT levels (all *P* > 0.05). The details are presented in Table 1.

**Figure 2:**
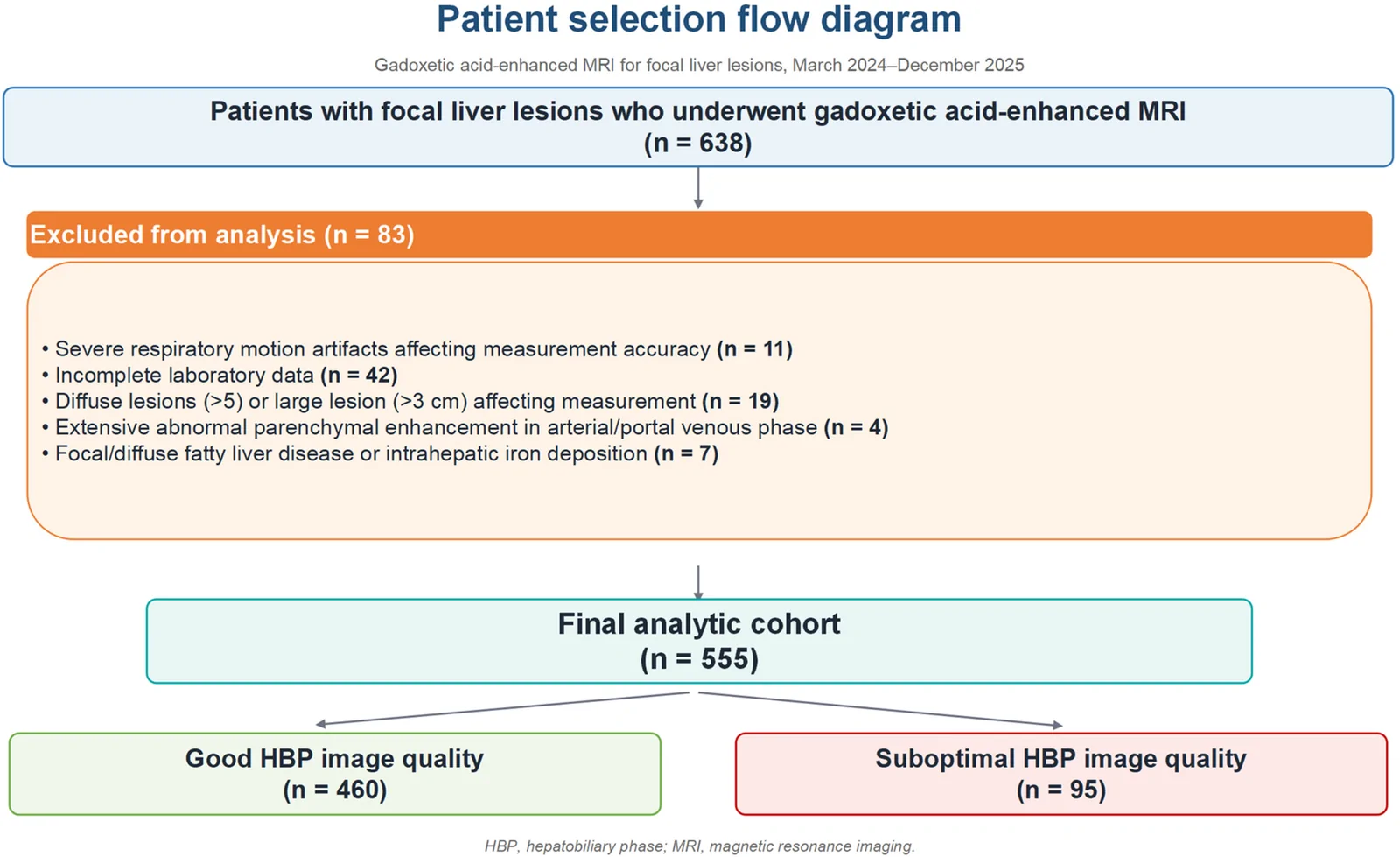
Participant flow diagram.

**Table 1:** Comparison of baseline characteristics, liver function scores, and laboratory indicators between the two groups.

Variable	Good image quality group (*n* = 460)	Suboptimal image quality group (*n* = 95)	Statistic	*P* value
Age (years)	53.69 ± 11.19	56.48 ± 10.49	−2.236	0.026^a^
Sex			0.578	0.447^c^
Male	326 (70.87%)	71 (74.74%)		
Female	134 (29.13%)	24 (25.26%)		
BMI category(kg/m^2^)			2.373	0.018^b^
Underweight＜18.5	32 (6.96%)	11 (11.58%)		
Normal weight 18.5–23.9	253 (55.00%)	58 (61.05%)		
Overweight 24–27.9	145 (31.52%)	24 (25.26%)		
Obesity ≥28	30 (6.52%)	2 (2.11%)		
Smoking history			0.552	0.458^c^
Yes	156 (33.91%)	36 (37.89%)		
No	304 (66.09%)	59 (62.11%)		
Long-term heavy alcohol consumption			2.775	0.096^c^
Yes	130 (28.26%)	35 (36.84%)		
No	330 (71.74%)	60 (63.16%)		
History of previous gadoxetic acid-enhanced MRI examination			0.064	0.800^c^
Yes	239 (51.96%)	48 (50.53%)		
No	221 (48.04%)	47 (49.47%)		
Tumor history			11.319	0.010^c^
Hepatocellular carcinoma	264 (57.39%)	53 (55.79%)		
Cholangiocarcinoma	14 (3.04%)	10 (10.53%)		
Metastatic tumor	164 (35.65%)	30 (31.58%)		
Other benign lesions	18 (3.91%)	2 (2.11%)		
Liver cirrhosis			0.030	0.863^c^
Yes	170 (36.96%)	36 (37.89%)		
No	290 (63.04%)	59 (62.11%)		
Hepatitis B virus infection			2.063	0.151^c^
Yes	255 (55.43%)	45 (47.37%)		
No	205 (44.57%)	50 (52.63%)		
COPD			0.729	0.393^c^
Yes	49 (10.65%)	13 (13.68%)		
No	411 (89.35%)	82 (86.32%)		
Diabetes mellitus			1.832	0.176^c^
Yes	46 (10.00%)	14 (14.74%)		
No	414 (90.00%)	81 (85.26%)		
Hypertension			1.813	0.178^c^
Yes	71 (15.43%)	20 (21.05%)		
No	389 (84.57%)	75 (78.95%)		
ALBI score	−2.57 (−2.81, −2.32)	−2.16 (−2.53, −1.71)	6.582	<0.001^b^
MELD score	7.02 (6.43, 8.00)	8.00 (6.71, 11.68)	5.180	<0.001^b^
TBIL（μmol/L）	12.50 (9.07, 16.75)	16.20 (10.65, 29.90)	4.203	<0.001^b^
GGT(U/L)	46.00 (25.75, 92.00)	79.00 (42.50, 245.50)	4.729	<0.001^b^
INR	1.01 (0.96, 1.08)	1.06 (0.97, 1.17)	2.660	0.008^b^
PLT(×10^9^/L)	196.00 (142.75, 262.25)	181.00 (121.50, 270.50)	1.057	0.291^b^
TBA（μmol/L）	7.00 (3.80, 13.65)	12.50 (5.80, 34.20)	4.508	<0.001^b^
DBIL（μmol/L）	4.60 (3.27, 6.20)	7.00 (4.30, 14.45)	5.742	<0.001^b^
IBIL（μmol/L）	7.85 (5.70, 10.62)	8.70 (6.45, 13.90)	2.340	0.019^b^
ALP(U/L)	85.00 (66.00, 116.25)	119.00 (83.50, 177.00)	5.541	<0.001^b^
Cr（μmol/L）	72.00 (61.00, 84.00)	68.00 (60.00, 83.00)	1.105	0.269^b^
AST(U/L)	31.00 (25.00, 45.00)	38.00 (28.00, 65.50)	3.603	<0.001^b^
ALT(U/L)	24.00 (16.00, 39.00)	26.00 (19.50, 44.00)	1.642	0.100^b^
Hb(g/L)	129.00 (113.00, 143.00)	118.00 (96.50, 133.00)	4.532	<0.001^b^
ALB(g/L)	38.90 (36.10, 41.50)	35.10 (30.55, 38.65)	5.964	<0.001^b^
SF(μg/L)	241.50 (117.75, 475.25)	383.00 (179.50, 634.00)	3.042	0.002^b^

Normally distributed continuous variables are expressed as the mean ± standard deviation, non-normally distributed continuous variables are expressed as M (P25, P75), and categorical variables are expressed as *n* (%). ᵃCalculated using the independent-samples t test; ᵇcalculated using the Mann–Whitney U test; ᶜcalculated using Pearson's chi-square test.

BMI, body mass index; COPD, chronic obstructive pulmonary disease; ALBI, albumin-bilirubin score; MELD, Model for End-Stage Liver Disease score; TBIL, total bilirubin; GGT, gamma-glutamyl transferase; INR, international normalized ratio; PLT, platelet count; TBA, total bile acid; DBIL, direct bilirubin; IBIL, indirect bilirubin; ALP, alkaline phosphatase; Cr, creatinine; AST, aspartate aminotransferase; ALT, alanine aminotransferase; Hb, hemoglobin; ALB, albumin; SF, serum ferritin.

### 3.2 Distribution of subjective image-quality scores and interobserver agreement

The distribution of the subjective image-quality scores is summarized in Supplementary Table S1. For liver parenchymal signal intensity, scores of 1, 2, and 3 were assigned to 476 (85.8%), 64 (11.5%), and 15 (2.7%) patients, respectively. For common bile duct enhancement, the corresponding numbers were 508 (91.5%), 11 (2.0%), and 36 (6.5%). Overall, 460 patients (82.9%) had a score of 1 for both components and were classified as having good image quality, whereas 95 patients (17.1%) were classified as having suboptimal image quality. Among the 95 patients with suboptimal imaging, 48 (50.5%) had an abnormal liver parenchymal component only, 16 (16.8%) had an abnormal bile duct component only, and 31 (32.6%) had abnormalities in both components. Thus, 79 of 95 patients (83.2%) had a suboptimal liver parenchymal component and 47 of 95 (49.5%) had a suboptimal bile duct component. Interobserver agreement was high for both ordinal image-quality components. The quadratic weighted kappa was 0.922 (95% CI: 0.879–0.956) for liver parenchymal signal intensity, with an overall agreement of 96.8%, and 0.945 (95% CI: 0.913–0.972) for common bile duct enhancement, with an overall agreement of 97.3% (both *P* < 0.001; Table 2).

**Table 2:** Interobserver agreement in subjective hepatobiliary-phase image-quality assessment.

Image-quality component	Weighted *κ*	95% CI	Agreement (%)	*P* value
Liver parenchymal signal score	0.922	0.879–0.956	96.8	<0.001
Common bile duct enhancement score	0.945	0.913–0.972	97.3	<0.001

Weighted κ represents quadratic weighted Cohen's kappa. CI, confidence interval.

### 3.3 Correlations between liver function scores, laboratory indicators, and MRI image quality

Spearman's correlation analysis was used to evaluate the relationships between liver function scores, laboratory indicators, and image quality of gadoxetic acid-enhanced MRI. The results showed that LPC was negatively correlated with the ALBI and MELD scores and TBIL, GGT, TBA, DBIL, ALP, AST, and SF levels (all *P* < 0.05), and positively correlated with Hb and ALB (both *P* < 0.001). No significant correlations were observed between LPC and INR, PLT, IBIL, Cr, or ALT levels (all *P* ≥ 0.05).

The liver parenchymal signal score was positively correlated with the ALBI score, MELD score and TBIL, GGT, INR, TBA, DBIL, IBIL, ALP, AST, and SF levels (all *P* < 0.05), and negatively correlated with Hb and ALB levels (both *P* < 0.001). No significant correlations were observed between the liver parenchymal signal score and PLT, Cr, or ALT levels (all *P* ≥ 0.05).

The common bile duct enhancement score positively correlated with the ALBI score and MELD score and TBIL, GGT, INR, TBA, DBIL, IBIL, ALP, AST, ALT, and SF levels (all *P* < 0.05) and negatively correlated with Hb and ALB levels (all *P* < 0.05). No significant correlations were observed between the common bile duct enhancement score and PLT or Cr levels (all *P* ≥ 0.05). Despite statistical significance for several associations, the correlation coefficients were generally small in magnitude. Most correlations were weak (|r| < 0.3), whereas only the correlations of LPC with the ALBI score (r = −0.331) and ALB (r = 0.321) reached the prespecified moderate range. The details are presented in Table 3 and Figure 3.

**Table 3:** Correlation analysis between liver function scores, blood indicators, and imaging quality of gadoxetic acid–enhanced MRI.

Indicator	LPC		Liver parenchymal signal score		Common bile duct enhancement score	
	*r*	*P value*	*r*	*P value*	*r*	*P value*
ALBI score	−0.331	<0.001	0.282	<0.001	0.197	<0.001
MELD score	−0.097	0.023	0.203	<0.001	0.192	<0.001
TBIL	−0.126	0.003	0.165	<0.001	0.154	<0.001
GGT	−0.085	0.046	0.171	<0.001	0.218	<0.001
INR	−0.03	0.482	0.095	0.025	0.147	<0.001
PLT	0.068	0.112	−0.083	0.050	−0.007	0.866
TBA	−0.114	0.007	0.199	<0.001	0.153	<0.001
DBIL	−0.161	<0.001	0.22	<0.001	0.208	<0.001
IBIL	−0.062	0.147	0.095	0.025	0.086	0.043
ALP	−0.224	<0.001	0.222	<0.001	0.234	<0.001
Cr	0.065	0.127	−0.063	0.138	−0.053	0.211
AST	−0.101	0.018	0.165	<0.001	0.159	<0.001
ALT	0.055	0.193	0.069	0.105	0.105	0.013
Hb	0.275	<0.001	−0.202	<0.001	−0.122	0.004
ALB	0.321	<0.001	−0.269	<0.001	−0.159	<0.001
SF	−0.095	0.025	0.086	0.043	0.124	0.003

LPC, liver parenchyma-to-portal vein contrast ratio; ALBI, albumin-bilirubin score; MELD, Model for End-Stage Liver Disease score; TBIL, total bilirubin; GGT, gamma-glutamyl transferase; INR, international normalized ratio; PLT, platelet count; TBA, total bile acid; DBIL, direct bilirubin; IBIL, indirect bilirubin; ALP, alkaline phosphatase; Cr, creatinine; AST, aspartate aminotransferase; ALT, alanine aminotransferase; Hb, hemoglobin; ALB, albumin; SF, serum ferritin.

**Figure 3:**
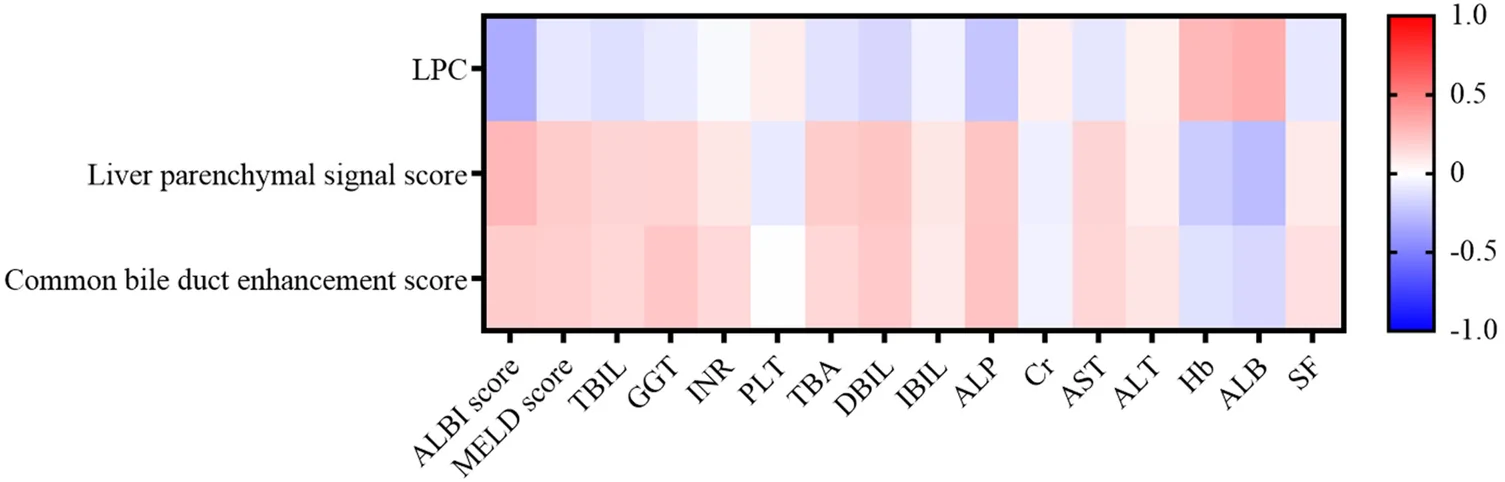
Correlation heatmap of liver function scores, laboratory indicators, and MRI image quality. LPC, liver parenchyma-to-portal vein contrast ratio; ALBI, albumin-bilirubin score; MELD, Model for End-Stage Liver Disease score; TBIL, total bilirubin; GGT, gamma-glutamyl transferase; INR, international normalized ratio; PLT, platelet count; TBA, total bile acid; DBIL, direct bilirubin; IBIL, indirect bilirubin; ALP, alkaline phosphatase; Cr, creatinine; AST, aspartate aminotransferase; ALT, alanine aminotransferase; Hb, hemoglobin; ALB, albumin; SF, serum ferritin.

### 3.4 Multicollinearity assessment, multivariable modeling, and sensitivity analysis

Multicollinearity assessment of the candidate predictor set identified substantial structural collinearity among several liver function measures. In particular, the VIFs were 51.125 for the ALBI score and 38.585 for ALB, reflecting the mathematical dependence of the ALBI score on albumin and bilirubin. Elevated VIFs were also observed for MELD (9.882), AST (9.853), TBIL (5.052), and IBIL (5.716). In contrast, no meaningful multicollinearity was observed among the four predictors retained in the final model, with VIFs of 1.543 for the ALBI score, 1.178 for GGT, 1.191 for TBA, and 1.226 for Hb. The final stepwise logistic regression model retained the ALBI score, GGT, TBA, and Hb, all of which were independently associated with suboptimal hepatobiliary phase imaging (all *P* < 0.05; Table 4). Detailed multicollinearity diagnostics for the candidate predictors are provided in Supplementary Table S2. In the sensitivity analysis additionally adjusted for age, BMI category, tumor history, liver cirrhosis, and hepatitis B virus infection, the associations of all four predictors remained statistically significant and directionally consistent with those in the primary model. The adjusted ORs were 2.335 (95% CI: 1.264–4.314; *P* = 0.007) for the ALBI score, 1.002 (95% CI: 1.001–1.004; *P* = 0.008) for GGT, 1.010 (95% CI: 1.003–1.017; *P* = 0.006) for TBA, and 0.984 (95% CI: 0.972–0.997; *P* = 0.013) for Hb. The adjusted model yielded an AUC of 0.777, and the direction and statistical significance of the four primary associations remained stable after adjustment for these available clinical covariates, including BMI category (Supplementary Table S3).

**Table 4:** Multivariate logistic regression analysis for predicting suboptimal hepatobiliary phase imaging in gadoxetic acid-enhanced MRI.

Variable	*B*	*SE*	*Wald*	*P*	*OR*	95% CI
ALBI score	0.872	0.286	9.268	0.002	2.391	1.364–4.19
GGT	0.003	0.001	9.12	0.003	1.003	1.001–1.004
TBA	0.01	0.004	8.19	0.004	1.01	1.003–1.017
Hb	−0.018	0.006	9.902	0.002	0.982	0.971–0.993
Constant	2.178	0.77	8.003	0.005	8.833	

ALBI, albumin-bilirubin score; GGT, gamma-glutamyl transferase; TBA, total bile acid; Hb, hemoglobin; OR, odds ratio; CI, confidence interval.

### 3.5 Discrimination and potential clinical utility of the combined model

ROC analysis showed AUCs of 0.714 (95% CI: 0.652–0.777) for the ALBI score, 0.654 (95% CI: 0.591–0.717) for GGT, 0.647 (95% CI: 0.581–0.713) for TBA, 0.648 (95% CI: 0.587–0.709) for Hb, and 0.758 (95% CI: 0.701–0.816) for the combined model. Pairwise DeLong tests showed that the AUC of the combined model was significantly higher than those of the ALBI score (*P* = 0.017), GGT (*P* = 0.002), TBA (*P* = 0.002), and Hb (*P* < 0.001). The optimal cutoff values determined by the maximum Youden index were −2.450 for the ALBI score, 139.5 U/L for GGT, 12.35 μmol/L for TBA, 124.5 g/L for Hb, and 0.176 for the predicted probability of the combined model. At the cutoff of 0.176, the combined model had a sensitivity of 63.16%, specificity of 78.70%, and Youden index of 0.419 (Table 5 and Figure 4A).

**Table 5:** ROC performance of individual predictors and the combined model for suboptimal hepatobiliary-phase imaging.

Predictor	AUC (95% CI)	Optimal cutoff	Sensitivity (%)	Specificity (%)	Youden index	*P* value*
ALBI score	0.714 (0.652–0.777)	>−2.450	70.53	64.57	0.351	0.017
GGT	0.654 (0.591–0.717)	>139.5	37.89	84.78	0.227	0.002
TBA	0.647 (0.581–0.713)	>12.35	52.63	71.96	0.246	0.002
Hb	0.648 (0.587–0.709)	≤124.5	62.11	60.22	0.223	<0.001
Combined model	0.758 (0.701–0.816)	>0.176	63.16	78.70	0.419	—

AUC, area under the curve; CI, confidence interval; ALBI, albumin–bilirubin; GGT, gamma-glutamyl transferase; TBA, total bile acid; Hb, hemoglobin. Optimal cutoff values were determined by maximizing the Youden index. *P values were obtained using paired DeLong tests comparing the AUC of the combined model with that of each individual predictor.

**Figure 4:**
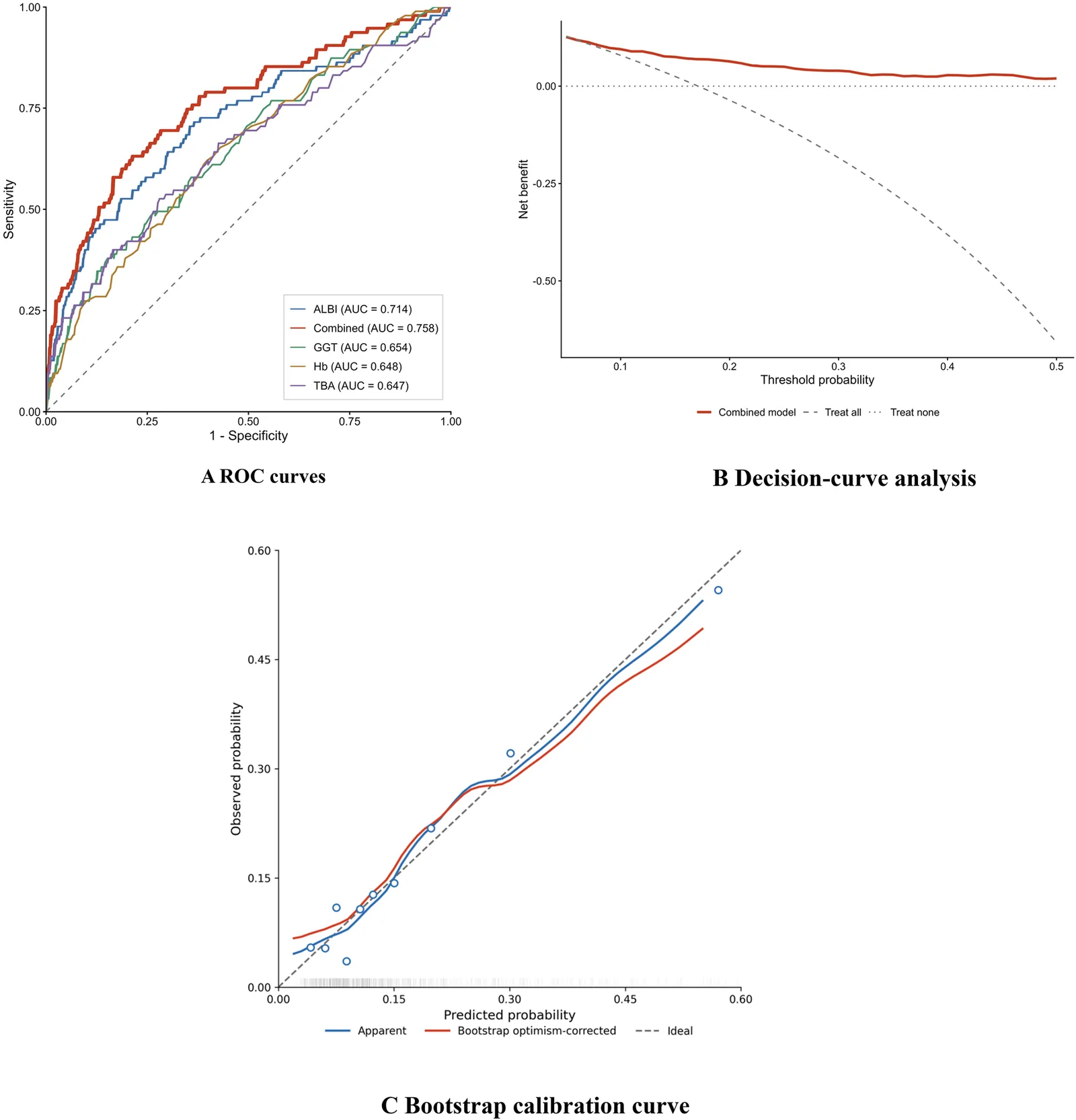
Discrimination, calibration, and potential clinical utility of the combined model for predicting suboptimal hepatobiliary-phase imaging. ALBI, albumin-bilirubin score; GGT, gamma-glutamyl transferase; TBA, total bile acid; Hb, hemoglobin; AUC, area under the curve. **(A)** Receiver operating characteristic (ROC) curves for the ALBI score, GGT, TBA, Hb, and the combined model. The combined model achieved an AUC of 0.758 (95% CI: 0.701–0.816). **(B)** Decision-curve analysis of the combined model. The model provided a greater net benefit than both the treat-all and treat-none strategies across evaluated threshold probabilities from 0.07 to 0.50.**(C)** Calibration plot of the combined model based on 1,000 bootstrap resamples in which the complete model-development procedure, including univariable screening and stepwise variable selection, was repeated. The apparent calibration curve and bootstrap optimism-corrected calibration curve are shown relative to the ideal 45° reference line.

The final logistic regression equation was: logit(P) = 2.178477 + 0.871562 × ALBI score + 0.002595 × GGT + 0.010054 × TBA−0.018360 × Hb, where P represents the predicted probability of suboptimal hepatobiliary-phase imaging. Accordingly, *P* = exp[logit(P)]/{1 + exp[logit(P)]}. The moderate sensitivity indicates that the model would not identify all patients with suboptimal hepatobiliary-phase imaging and should therefore not be considered a stand-alone screening tool. Decision-curve analysis showed that, among the evaluated threshold probabilities, the combined model provided a greater net benefit than both the treat-all and treat-none strategies across thresholds from 0.07 to 0.50 (Figure 4B).

### 3.6 Internal validation and calibration of the combined model

Bootstrap internal validation of the entire model-development procedure with 1,000 resamples showed a mean AUC optimism of 0.044. The apparent AUC of 0.758 yielded an optimism-corrected AUC of 0.714. Although lower than the apparent estimate, the corrected AUC remained above 0.70, indicating that the model retained moderate discriminatory ability after accounting for optimism associated with the complete model-development process. The apparent Brier score was 0.116 and the optimism-corrected Brier score was 0.128. The bootstrap optimism-corrected calibration curve showed some additional deviation from the ideal reference line at higher predicted probabilities (Figure 4C).

## 4 Discussion

This retrospective study investigated the relationships among laboratory indicators, liver function scores, and hepatobiliary phase image quality on Gd-EOB-DTPA-enhanced MRI and further evaluated their predictive value for suboptimal hepatobiliary phase imaging. Correlation analysis identified statistically significant associations between LPC and several liver function scores and laboratory indicators; however, these associations were predominantly weak in magnitude, with only the correlations of LPC with the ALBI score and ALB reaching the moderate range. This overall pattern is consistent with previous reports linking impaired liver function and laboratory abnormalities to reduced hepatobiliary-phase enhancement or suboptimal image quality (7, 12). Multivariate logistic regression analysis showed that the ALBI score and GGT, TBA, and Hb levels were independently associated with suboptimal hepatobiliary phase imaging during gadoxetic acid-enhanced MRI. The combined model showed moderate apparent discrimination for suboptimal hepatobiliary-phase imaging and performed better than the individual indicators evaluated in this cohort. When the entire model-development procedure, including univariable screening and stepwise selection, was repeated within each bootstrap sample, the AUC decreased from 0.758 to an optimism-corrected value of 0.714, while the Brier score increased from 0.116 to 0.128. Although the correction attenuated apparent performance, the optimism-corrected AUC remained above 0.70, suggesting that the model retained moderate discrimination after accounting for model-development optimism. The corrected calibration curve showed some additional deviation from the ideal line at higher predicted probabilities. Overall, these findings indicate some optimism in the apparent performance while supporting retained discriminatory ability after correction. Nevertheless, these analyses represent internal validation only and do not establish the transportability of the model to other populations or MRI practice settings. These findings support its potential value for pre-MRI risk stratification, although its performance does not support its use as a stand-alone clinical decision tool.

Gd-EOB-DTPA is a hepatobiliary-specific contrast agent, and its hepatobiliary phase enhancement depends mainly on hepatocyte uptake of the contrast agent and subsequent biliary excretion (18, 19). As a quantitative indicator reflecting liver parenchymal enhancement relative to the portal vein, LPC can be used to evaluate the degree of liver parenchymal enhancement during the hepatobiliary phase to a certain extent (20). A higher LPC indicates a greater liver parenchymal enhancement during the hepatobiliary phase, whereas a lower LPC may suggest impaired hepatocyte uptake or excretion of the contrast agent. Correlation analysis showed that LPC was negatively correlated with ALBI score, MELD score and TBIL, GGT, TBA, DBIL, ALP, AST, and SF levels, and positively correlated with Hb and ALB levels. Although the directions of these correlations were consistent with an association between poorer hepatic function and reduced hepatobiliary-phase enhancement, their generally weak magnitudes indicate that no single laboratory indicator closely reflects hepatobiliary-phase enhancement. This may be because the hepatic uptake of Gd-EOB-DTPA depends on OATPs located on the hepatocyte membrane, followed by excretion through the biliary system. When patients have impaired hepatocyte function, cholestasis, or elevated bilirubin levels, the uptake and excretion of the contrast agent may be affected, resulting in reduced hepatobiliary phase signal intensity (21). ALB is an important indicator of hepatic synthetic function and nutritional status. A higher ALB level generally suggests a better hepatic functional reserve; therefore, the uptake capacity of Gd-EOB-DTPA may be relatively stronger, resulting in more sufficient hepatobiliary phase enhancement. Previous studies have shown that hepatocyte uptake and T1 mapping parameters derived from Gd-EOB-DTPA-enhanced MRI are correlated with ALBI grade, suggesting that hepatobiliary phase imaging parameters can reflect hepatic functional reserve to a certain extent (22). Hb was positively correlated with LPC, indicating that patients with higher Hb levels tended to have better liver parenchymal enhancement during the hepatobiliary phase. This may be related to a patient's overall nutritional status, chronic wasting, hepatic functional reserve, or general physical condition. Previous studies have suggested that anemia is common in patients with liver cirrhosis and chronic liver disease and may reflect disease severity, nutritional status, and systemic consumption (23). However, Hb is not directly involved in the uptake or excretion of Gd-EOB-DTPA, and the specific mechanism underlying the relationship between Hb and hepatobiliary phase image quality requires further investigation. It should be noted that the correlation coefficients between these indicators and LPC in this study were generally within the weak to low-moderate range, suggesting that the explanatory power of a single laboratory indicator for hepatobiliary phase image quality is limited. Hepatobiliary phase image quality may be affected by multiple factors, including hepatocyte function, biliary excretion, hepatic perfusion, underlying liver disease, and systemic nutritional status. Therefore, relying on a single indicator to evaluate image quality has certain limitations, and predictions based on a combination of multiple indicators may be more consistent with clinical practice (24).

Multivariate logistic regression analysis showed that the ALBI score and GGT, TBA, and Hb levels were independently associated with suboptimal hepatobiliary phase imaging during Gd-EOB-DTPA-enhanced MRI. The ALBI score is calculated based on TBIL and ALB levels and can objectively reflect the hepatic functional reserve without relying on subjective clinical indicators such as ascites or hepatic encephalopathy (25). In this study, an increased ALBI score was associated with an increased risk of suboptimal hepatobiliary phase imaging. This may be because a higher ALBI score indicates elevated bilirubin or decreased albumin levels, reflecting the impaired synthetic, metabolic, and excretory functions of hepatocytes. Consequently, the uptake capacity of Gd-EOB-DTPA may decrease, leading to insufficient liver parenchymal enhancement during the hepatobiliary phase. Previous studies have shown that the ALBI grade is correlated with functional liver imaging scores derived from Gd-EOB-DTPA-enhanced MRI, suggesting that the ALBI score may indirectly reflect hepatobiliary phase image quality (26). Therefore, the ALBI score may not only be used for liver function assessment but also serve as an important indicator for predicting hepatobiliary phase image quality in Gd-EOB-DTPA-enhanced MRI. GGT and TBA levels were also independently associated with suboptimal hepatobiliary-phase imaging in this study. GGT mainly reflects biliary tract injury and cholestasis, whereas TBA is closely associated with bile acid metabolism and hepatocyte uptake, transport, and excretion. Previous studies have indicated that bile acid metabolism and related signaling pathways are closely associated with the progression of various liver diseases, and the disruption of bile acid homeostasis may reflect cholestasis and abnormal hepatocellular transport function (27). In this study, elevated GGT and TBA levels were associated with an increased risk of suboptimal hepatobiliary phase imaging. A possible mechanism is that the hepatocellular transport of bile acids and organic anions decreases when cholestasis or biliary excretion disorder occurs. Gd-EOB-DTPA may share competitive or commonly impaired transport pathways with substances such as bilirubin and bile acids during hepatocyte uptake and biliary excretion, ultimately leading to insufficient hepatobiliary excretion of the contrast agent, decreased liver parenchymal enhancement, and poor bile duct visualization (28). Therefore, GGT and TBA not only reflect the degree of cholestasis but may also indirectly indicate the risk of reduced hepatobiliary phase image quality in Gd-EOB-DTPA-enhanced MRI. In addition, this study found that Hb was independently associated with suboptimal hepatobiliary-phase imaging, with an OR below 1, indicating that higher Hb levels were associated with a lower likelihood of suboptimal imaging. Hb is not traditionally considered a direct indicator of hepatocyte contrast agent uptake capacity; however, to some extent, it may reflect the anemia status, nutritional status, chronic tumor-related consumption, and general physical condition of the patient. Patients with lower Hb levels may have poorer systemic reserves or a greater chronic disease burden, which may be associated with decreased hepatic functional reserves, altered hepatic perfusion, or weakened metabolic function. In this study, Hb levels positively correlated with LPC, suggesting that patients with higher Hb levels tended to have better liver parenchymal enhancement during the hepatobiliary phase. However, no direct causal relationship was observed between Hb and Gd-EOB-DTPA uptake or excretion. The specific pathophysiological mechanism by which Hb levels act as a predictive factor requires further clarification using larger sample sizes and prospective studies.

In this study, the combined model incorporating the ALBI score, GGT, TBA, and Hb achieved moderate apparent discrimination for suboptimal hepatobiliary-phase imaging, with an AUC of 0.758. Pairwise DeLong comparisons showed that its apparent discrimination was significantly greater than that of each individual predictor. However, at the Youden-derived probability cutoff of 0.176, the sensitivity was 63.16%, indicating that a substantial proportion of patients with suboptimal imaging would remain unidentified if the model were used alone. Bootstrap validation of the complete model-development procedure yielded an optimism-corrected AUC of 0.714 and an optimism-corrected Brier score of 0.128. Although this confirmed some optimism in the apparent performance, the corrected AUC remained above 0.70, supporting retained moderate discrimination after accounting for the full model-development process. The optimism-corrected calibration curve showed some additional deviation from the ideal line at higher predicted probabilities. Decision-curve analysis showed a greater net benefit than the treat-all and treat-none strategies across evaluated threshold probabilities from 0.07 to 0.50. Collectively, these findings support the model as a preliminary adjunct for pre-MRI risk stratification rather than a stand-alone screening or decision tool. Although identification of patients at increased risk of suboptimal hepatobiliary-phase imaging could ultimately help inform acquisition planning, the present study does not establish that model-guided modification of MRI protocols improves image quality or patient outcomes. Independent external validation and prospective evaluation of model-guided imaging strategies are therefore required before clinical implementation. Previous studies have nevertheless suggested that hepatobiliary-phase acquisition may benefit from adjustment according to liver function status (29), providing a rationale for prospective evaluation of model-guided imaging strategies.

This study had several limitations. First, this was a single-center retrospective study, and selection bias cannot be excluded. When the complete model-development procedure was repeated within each bootstrap sample, the optimism-corrected AUC was 0.714 compared with the apparent AUC of 0.758, indicating some model-development optimism associated with univariable screening and stepwise variable selection. However, the corrected AUC remained above 0.70, supporting retained moderate discrimination after internal correction. The model was developed and internally validated within the same cohort and has not undergone independent external validation. Its transportability and performance across different institutions, patient populations, and MRI practice settings therefore remain uncertain and should be evaluated in independent multicenter and preferably prospective cohorts before clinical implementation. Second, the binary classification of good versus suboptimal hepatobiliary-phase image quality was based on an operational composite definition requiring optimal scores for both liver parenchymal enhancement and common bile duct enhancement. Although both domains are established features of hepatobiliary-phase assessment (6, 17), this specific composite threshold has not been independently validated as a lesion-specific measure of diagnostic adequacy. Moreover, biliary excretion primarily reflects hepatobiliary transport function and may not contribute equally to focal-lesion assessment across all clinical indications. Therefore, the present findings should be interpreted as relating to overall hepatobiliary-phase image quality as operationally defined in this study rather than to a universally validated threshold for diagnostic adequacy. Although the two radiologists showed high interobserver agreement based on quadratic weighted kappa, subjective image-quality assessment may still be influenced by reader experience and does not eliminate the inherent limitations of observer-dependent scoring. Third, the study population was selected using imaging-based exclusion criteria intended to reduce factors that could interfere with quantitative and subjective hepatobiliary-phase assessment. In particular, patients with severe respiratory motion artifacts, fatty liver disease, or intrahepatic iron deposition were excluded because these conditions can independently alter image quality or hepatic signal intensity. However, these conditions are encountered in routine clinical practice and can themselves contribute to suboptimal imaging. Their exclusion therefore limits the generalizability of the present findings, particularly to patients with substantial steatosis, iron deposition, or motion-related image degradation. The performance of the model in these clinically relevant populations requires separate validation. In addition, detailed information on previous treatments and other underlying hepatic disease characteristics was not consistently available in the retrospective dataset and could not be fully accounted for in the adjusted analysis. Residual confounding from these factors therefore cannot be excluded. Finally, the predictive model used in this study was established based only on routine laboratory indicators and liver function scores. Other indicators that may reflect the hepatic functional reserve, such as ICG-R15, ICG-K, liver volume, liver stiffness, and quantitative MRI parameters, were not included. Future studies should expand the sample size, incorporate additional quantitative imaging parameters and liver function assessment indicators, and establish more stable and accurate predictive models.

## 5 Conclusion

In conclusion, the ALBI score, GGT, TBA, and Hb were independently associated with suboptimal hepatobiliary-phase imaging during Gd-EOB-DTPA-enhanced MRI. The combined model showed moderate discrimination and may provide preliminary support for pre-MRI risk stratification. However, its clinical utility and ability to guide individualized imaging protocols require further evaluation and external validation.

## Data Availability

The original contributions presented in the study are included in the article/Supplementary Material, further inquiries can be directed to the corresponding author.
