# Supplementary material for: Laboratory indicators combined with liver function scores for predicting suboptimal hepatobiliary phase imaging during gadoxetic acid-enhanced MRI
Source: Front Med (Lausanne). 2026 Oct 13;13:1945459. doi: 10.3389/fmed.2026.1945459 (PMC13710012; doi:10.3389/fmed.2026.1945459)
Supplement: Supplementary file 1 [file Table1.docx]

**Supplementary Table S1. Distribution of subjective hepatobiliary-phase image-quality scores and components contributing to the composite classification**

**Panel A. Distribution of individual image-quality scores**

| Image-quality component | Score | *n* (%) |
| --- | --- | --- |
| Liver parenchymal signal intensity | 1 | 476 (85.8) |
|  | 2 | 64 (11.5) |
|  | 3 | 15 (2.7) |
| Common bile duct enhancement | 1 | 508 (91.5) |
|  | 2 | 11 (2.0) |
|  | 3 | 36 (6.5) |

**Panel B. Components contributing to the composite image-quality classification**

| Classification | *n* (%) |
| --- | --- |
| Good image quality: both components scored 1 | 460 (82.9) |
| Suboptimal: liver parenchymal component only | 48 (8.6) |
| Suboptimal: bile duct component only | 16 (2.9) |
| Suboptimal: both components | 31 (5.6) |
| Total suboptimal image quality | 95 (17.1) |

**Note:** Good image quality was operationally defined as a score of 1 for both liver parenchymal signal intensity and common bile duct enhancement. All remaining combinations were classified as suboptimal image quality. Percentages are calculated using the total cohort (n = 555).

**Supplementary Table S2. Multicollinearity diagnostics for candidate predictors and predictors retained in the final model**

**Panel A. Multicollinearity diagnostics for candidate predictors included in the primary multivariable modeling process**

| Predictor | VIF/GVIF | Df | Tolerance |
| --- | --- | --- | --- |
| ALBI score | 51.125 | 1 | 0.020 |
| MELD score | 9.882 | 1 | 0.101 |
| TBIL | 5.052 | 1 | 0.198 |
| GGT | 2.685 | 1 | 0.372 |
| INR | 3.224 | 1 | 0.310 |
| TBA | 1.454 | 1 | 0.688 |
| DBIL | 2.506 | 1 | 0.399 |
| IBIL | 5.716 | 1 | 0.175 |
| ALP | 2.466 | 1 | 0.406 |
| AST | 9.853 | 1 | 0.101 |
| Hb | 1.752 | 1 | 0.571 |
| ALB | 38.585 | 1 | 0.026 |
| SF | 1.385 | 1 | 0.722 |
| Age | 1.324 | 1 | 0.755 |
| BMI category | 1.492 | 3 | — |
| Tumor history | 3.019 | 3 | — |

**Panel B. Multicollinearity diagnostics for predictors retained in the final model**

| Predictor | VIF | Tolerance |
| --- | --- | --- |
| ALBI score | 1.543 | 0.648 |
| GGT | 1.178 | 0.849 |
| TBA | 1.191 | 0.839 |
| Hb | 1.226 | 0.815 |

**Note:** VIF, variance inflation factor; GVIF, generalized variance inflation factor; Df, degrees of freedom; ALBI, albumin–bilirubin; MELD, Model for End-Stage Liver Disease; TBIL, total bilirubin; GGT, gamma-glutamyl transferase; INR, international normalized ratio; TBA, total bile acid; DBIL, direct bilirubin; IBIL, indirect bilirubin; ALP, alkaline phosphatase; AST, aspartate aminotransferase; Hb, hemoglobin; ALB, albumin; SF, serum ferritin; BMI, body mass index. Candidate predictors were defined as variables showing statistically significant between-group differences in the univariable analyses (P < 0.05). For categorical predictors with more than one degree of freedom, GVIFs were calculated; the adjusted GVIFs [GVIF^(1/(2×Df))] were 1.069 for BMI category and 1.202 for tumor history. For single-degree-of-freedom predictors, conventional VIF and tolerance (1/VIF) are reported. Tolerance was not reported for multi-degree-of-freedom categorical predictors. A VIF >10 was considered indicative of substantial multicollinearity.

**Supplementary Table S3. Sensitivity analysis of the final four predictors after adjustment for potential clinical confounders**

| Variable | β | SE | OR | 95% CI | P value |
| --- | --- | --- | --- | --- | --- |
| ALBI score | 0.848 | 0.313 | 2.335 | 1.264–4.314 | 0.007 |
| GGT | 0.002 | 0.001 | 1.002 | 1.001–1.004 | 0.008 |
| TBA | 0.010 | 0.004 | 1.010 | 1.003–1.017 | 0.006 |
| Hb | −0.016 | 0.006 | 0.984 | 0.972–0.997 | 0.013 |

**Note:** The sensitivity model additionally adjusted for age, BMI category, tumor history, liver cirrhosis, and hepatitis B virus infection. The AUC of the adjusted model was 0.777. OR, odds ratio; CI, confidence interval; ALBI, albumin–bilirubin; GGT, gamma-glutamyl transferase; TBA, total bile acid; Hb, hemoglobin; BMI, body mass index.
